# Inflammogenesis of Secondary Spinal Cord Injury

**DOI:** 10.3389/fncel.2016.00098

**Published:** 2016-04-13

**Authors:** M. Akhtar Anwar, Tuqa S. Al Shehabi, Ali H. Eid

**Affiliations:** ^1^Department of Biological and Environmental Sciences, Qatar UniversityDoha, Qatar; ^2^Department of Health Sciences, Qatar UniversityDoha, Qatar; ^3^Department of Pharmacology and Toxicology, Faculty of Medicine, American University of BeirutBeirut, Lebanon

**Keywords:** inflammation, ischemia-reperfusion injury (IRI), spinal cord injury (SCI), reactive oxygen species (ROS), leukocytes, glia, therapeutics

## Abstract

Spinal cord injury (SCI) and spinal infarction lead to neurological complications and eventually to paraplegia or quadriplegia. These extremely debilitating conditions are major contributors to morbidity. Our understanding of SCI has certainly increased during the last decade, but remains far from clear. SCI consists of two defined phases: the initial impact causes primary injury, which is followed by a prolonged secondary injury consisting of evolving sub-phases that may last for years. The underlying pathophysiological mechanisms driving this condition are complex. Derangement of the vasculature is a notable feature of the pathology of SCI. In particular, an important component of SCI is the ischemia-reperfusion injury (IRI) that leads to endothelial dysfunction and changes in vascular permeability. Indeed, together with endothelial cell damage and failure in homeostasis, ischemia reperfusion injury triggers full-blown inflammatory cascades arising from activation of residential innate immune cells (microglia and astrocytes) and infiltrating leukocytes (neutrophils and macrophages). These inflammatory cells release neurotoxins (proinflammatory cytokines and chemokines, free radicals, excitotoxic amino acids, nitric oxide (NO)), all of which partake in axonal and neuronal deficit. Therefore, our review considers the recent advances in SCI mechanisms, whereby it becomes clear that SCI is a heterogeneous condition. Hence, this leads towards evidence of a restorative approach based on monotherapy with multiple targets or combinatorial treatment. Moreover, from evaluation of the existing literature, it appears that there is an urgent requirement for multi-centered, randomized trials for a large patient population. These clinical studies would offer an opportunity in stratifying SCI patients at high risk and selecting appropriate, optimal therapeutic regimens for personalized medicine.

## Introduction

Spinal cord injury (SCI) remains a major cause of disability (Singh et al., 2014). This debilitating event is a principal life-changer of previously-healthy individuals. Not only does it drain limited health-care budgets, but it also influences the psyche of the subjects. Likewise, SCI impacts the social demands put in place to ameliorate the well-being of the incapacitated individual. This is because it often involves immediate and extended family members and friends. Since the times of the Egyptian Pharaohs, SCI has been an area of neglect in relation to pharmacological research for the simple fact that it was considered to be terminal, and that surgery is the sole option (Silva et al., 2014). Currently, there is a realization that if rapid intervention is applied, then SCI is definitely treatable, and the SCI-associated high rates of mortality can be reduced (Garshick et al., 2005; Silva et al., 2014). This offers a glimmer of hope for patients and society at large. A consequential sequence of SCI is the significant neurological or psychological deficit, which obviously contributes to the overall burden of this condition. This results in reduced life span, loss of productive years, and steep health care costs. Since SCI is ranked as the most calamitous cause of morbidity, it does not come as surprising that the total costs are staggering. In Spain, $131 to 302 million for 2007 were spent to deal with this debilitation (García-Altés et al., 2012), whereas the outlay in Canada is estimated to be Canadian $2.7 billion for 1389 new patients per year (Krueger et al., 2013). The annual expenditure for American citizens suffering from SCI-related disabilities is in excess of $10 billion (Wilt et al., 2008).

Mention must be made of incidence of SCI that occurs as a complication arising from surgery for thoracic or abdominal aortic aneurysms during the procedure to repair the damaged aorta (Hollier et al., 1992). Prolonged aortic cross-clamping or ligation of intercostal or lumber arteries proceeds to spinal cord ischemia. This can affect the thoracic, lumbar or sacral regions, and may lead to paraplegia (hindlimb paralysis; Bicknell et al., 2009; Shimizu and Yozu, 2011). Victims of motor vehicle accidents, war- or combat-related injuries, frail elderly subjects who fall, and individuals with gunshot wounds may also suffer from paraplegia, or even quadriplegia attributable to neurological problems (Smith et al., 2005; Blair et al., 2012; Schoenfeld et al., 2013; Singh et al., 2014). Horseback riding (Lin et al., 2011), high contact sports like ice hockey, American football and rugby (scrummage, tackles and rucks) place players at risk of injuries to the cervical spine, which occur at 4th, 5th and 6th vertebrae with hyperextension, hyperflexion or axial loading (Quarrie et al., 2002; Rihn et al., 2009). As a consequence of the high impact collisions, the damages result in a spectrum of traumatic injuries, from complete recovery at one end, to partial/complete paraplegia or tetraplegia, to death at the other extreme end (Singh et al., 2014; Valparaiso et al., 2015).

Spinal cord injuries (SCI) can be classified into primary and secondary. Primary SCI often results from mechanical impaction to the spine, concomitant with or followed by compression, contusion, stretching or kinking of the spinal cord (Stahel et al., 2012; Table 1). Secondary SCIs refers to the multifaceted pathological mechanisms that start after primary SCIs and can last up to weeks (Wilson et al., 2013). These events include, but are not limited to, breakdown of blood-spinal cord barrier (BSCB), neuroinflammation, oxidative stress, neuronal injury, and ischemic dysfunction (Table 1). SCIs are also associated with other functional problems, including neuropathic pain and autonomic dysfunction, causing incontinence of bladder, rectum and anus (lower gastrointestinal tract), as well as impotence (Rosenzweig and McDonald, 2004; Singh et al., 2014). Therefore, the pathophysiology of SCI consists of a primary injury along with sequential secondary damages emanating from multi-cascade of patho-mechanisms. These secondary pathologic series of events lead to further damages to the SC.

**Table 1:** **Summary of temporal sequence of pathophysiological events during SCI phases, primary and secondary**.

Primary	Secondary		
Immediate (<2 h)	Acute (<2 days)	Intermediate (<2 weeks)	Chronic (weeks/months)
Mechanical injury: compression, contusion	Commencement of innate immunity: microglia activation and neutrophil infiltration	Full-blown inflammation: innate immune response (monocytes, macrophages M1, microglia M1 & reactive astrocytes) and adaptive immunity (T and B cells)	Wallerian degeneration: cytoskeleton disintegration, loss of cell membrane, axon fragmentation
Hemodynamic instability	Release of inflammatory mediators: pro-inflammatory cytokines (IL-1, IL-6, TNF-α IFN-γ) and chemokines (CXCL1, CXCL12)	Apoptosis of oligodendrocytes	Apoptosis of oligodendrocytes
Vasospasm	Oxidative stress: increased expression of iNOS; elevated levels of free radicals, ROS, RNS, and NO; increased lipid peroxidation	Demyelination	Demyelination: fragmentation of *myelin*, *cellular debris*
Reduced blood flow	Glutaminergic excitotoxicity: cell damage, depolarization	Initiation of cyst formation	Glial scar maturation
Hemorrhage	Metabolic derangement: ionic imbalance (Na^+^, K^+^, Cl^-^, Ca^2+^); acidosis; ATP, decreased O_2_ and glucose	Neuropathic pain	Cavitation
Edema	Mitochondrial damage: pore formation; *cyt c* release	Glial scar initiation	Lesion stabilization
Alteration of vascular structure	Cytoskeletal damage	Chemokine release: CXCL1, CXCL9, CXCL10, CXCL12	Chemokine release: CXCL12
Ischemic necrosis	Apoptosis	Phagocytosis: RBCs, myelin and neutrophils	Neuron growth inhibitors: Nogo, MAG, ROCK
Thrombosis	Demyelination	Resolution/Repair: resolution of edema; Repair of BSCB	Repair/ Recovery/ Resolution/ Regeneration neuronal sprouting, Regeneration of axon clusters, Complement- dependent, Neuro- reparatory processes, Change to anti-inflammatory phenotype of microglia and macrophages (M2)
Destruction of neural tissue	Neuronal cell death
Activation of microglia	Neurogenic shock
Axonal shearing	BSCB permeability
Neuronal cell death	Complement-activated neurodegradation
Myelin debris: release of DAMPs	Release of proteases: MMPs, calpain, caspases
Evidence of complement protein C3

*Mechanical-derived primary damage is followed by a prolonged secondary injury consisting of overlapping, different phases. This involves interplay of infiltrating leukocytes and residential parenchymal cells (adaptive and innate immunity), and their releasates (inflammatory cytokines and chemokines, anti-inflammatory cytokines)*.

Together, these bio-reactions drive a homeostatic imbalance leading to an inflammatory milieu that is linked to hemostatic and metabolic changes (Guízar-Sahagún et al., 2004) as well as hemodynamic alterations in the pathophysiology of SCI. In the present review, these will be highlighted, along with a prior description of available animal models for investigation.

## Animal Models for SCI

At the outset, it is important to realize that SCI in man is heterogeneous in nature. Diverse animal species (rats, mice, sheep, rabbits, dogs, opossums and baboons) have been used to simulate SCI. Compared to other models, the rat offers several advantages such as its moderate cost, vascular anatomy and more importantly, its physiology which is rather highly comparable to humans’. Moreover, the dimension of isolated arteries is manageable for investigating vascular function. The SC and surrounding tissue are of reasonable size. In a 280 gm rat, the total blood volume is almost 16 ml, and approximately 10 ml of blood can be obtained for hematological studies (Akhtar et al., 2008; Choo et al., 2008; Cheriyan et al., 2014).

**Figure 1:**
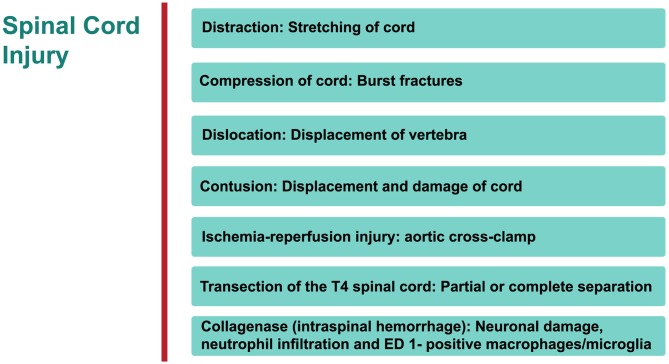
**An overview of rat models of Spinal cord injury (SCI).** The principle site of injury is the dorsal thoracic spine, and the dorsal spinal artery.

Specific therapeutic modalities have been applied to mitigate the induced SCI in animal models. However, to date, the translation of these promising outcomes from the lab to the bedside has been without success (Martirosyan et al., 2011; Varma et al., 2013). The type of SCI most frequently induced is dependent on the mechanical impact of the injury which can then be categorized into: compression, contusion, distraction, dislocation, transection, collagenase and ischemia-reperfusion (interruption of blood flow) injury (IRI; Choo et al., 2007, 2008, 2009; Akhtar et al., 2008; Rummery et al., 2010; Cheriyan et al., 2014; Losey and Anthony, 2014; Kato et al., 2015; Figure 1). Each of these models bears many advantages, but is not without some limitations (Akhtar et al., 2008; Choo et al., 2008; Rummery et al., 2010; Cheriyan et al., 2014; Losey and Anthony, 2014). For example, the ischemia-reperfusion SCI injury model may lead to aortic cross-clamping resulting in several complications. Some contusion models suffer from some weakness such as the clamping technique (in infinite horizon, force-controlled contusion) or imprecise duration of impact (in MASCIS weight-drop contusion approach). Many compression models may also suffer from the absence of recording injury parameters. Inconsistent reproducibility or uncommon clinical SCI type are major limitations of partial or complete transection models, respectively. It must be stressed that most human SCIs involve compressive and contusive injury. The aforementioned references elaborate on details of following models.

### Distraction

Cranial-caudal distraction between C4-C5, edema (no overt hemorrhage), axonal damage, demyelination, gliosis, rostrally-stretched nodes of Ranvier.

*Advantages/disadvantages*: in humans, a significant proportion of injuries are located in cervical region of the spine.

### Compression

Significant vascular injury (hemorrhagic necrosis, hypoperfusion), neuronal ischemia, neurological derangement. *Advantages/disadvantages*: one of the most commonly used procedures to investigate SCI.

### Dislocation

C4/C5 dorso-ventral dislocation, substantial axonal degeneration (axolemma stretched rostrally), deformation in node of Ranvier at epicenter of injury, vascular damage in gray matter, extended lesion in dorsal corticospinal tract, activated astrocytes and microglial (cells reactive bi-directionally: cranial to caudal), apoptosis (*cytochrome C* (*cyt c*) release from mitochondria in penumbra).

*Advantages/disadvantages*: again this technique examines the injury in cervical region of SC.

### Contusion

Widespread tissue pathology, extending both rostrally and caudally from epicenter of injury—disruption of white and gray matter, intraparenchymal hemorrhage, hypotension, bradycardia, cellular debris, diffuse axonal injury, glia activation with cavities and fluid-filled cysts, apoptosis of oligodendrocytes, macrophage infiltration and activated microglial at epicenter, distortion of nodes of Ranvier.

*Advantages/disadvantages*: the most widely used method for investigating SCI.

### Ischemia-Reperfusion Injury

Vascular derangement (ischemia, hypoxia, vasospasms, thrombosis), neuronal damage, lesion cavity.

*Advantages/disadvantages*: no mechanical trauma to SC, pathology not consistently reproducible.

### Transection of Spinal Cord

Tissue pathology focused at epicenter of injury—white matter apoptosis, demyelination, macrophage infiltration and microglia activation at epicenter.

*Advantages/disadvantages*: rare in humans; commonly used for examining degeneration and regeneration of axons.

### Intraspinal Hemorrhage (Collagenase)

Neutrophil infiltration and ED 1 positive macro-phages/microglia.

*Advantages/disadvantages*: not very common.

It is important to note that animal models of SCI pain have been developed to increase our understanding of depression (Luedtke et al., 2014) as well as the pain syndrome (Hao and Xu, 2003). Without a doubt, our knowledge and insight into SCI from investigations on these animal models has significantly increased during the last fifteen years. Nonetheless, a number of limitations have been acknowledged, including the site of injury. For example, in humans, the predominant location of injury is at an anterior position and the cervical spine is typically involved, whereas animal studies are based on the dorsal side and thoracic spine is functionally deteriorated. Another disadvantage of using rats is that corticospinal tract lesions are observed on the dorsal plane, and therefore disability is limited. Additionally, in humans, the anterior spinal artery, which transports blood to over 70% of SC tissue, is damaged or ruptured; on the other hand, the dorsal spinal artery is impinged upon in animal models of SCI (Akhtar et al., 2008). Further, laminectomy (invasive techniques which affect the experimental outcome), anesthesia (neuroprotective; Akhtar et al., 2008), and gender are all critical to well-planned studies. Specifically, when using female rats, the researcher needs to cater for the estrous cycle (metestrus, diestrus, proestrus and estrus), during which steroid hormones (estrogen and progesterone) are fluctuating (confounding elements), and the endocrines are considered to be neuroprotective. Therefore, the animals need to be synchronized to a specific phase of the estrous cycle, or to use ovariectomized animals. Contextually, although rodents are routinely used to simulate the injuries observed in the clinic, the non-human primate is undeniably more appropriate for human type of SCIs (Nesathurai et al., 2006; Akhtar et al., 2008; Nout et al., 2012).

Different animal models are being used to address multiple paradigms arising from the devastating trauma of SCI, with the ultimate aim of being able to ameliorate the suffering of human subjects. The use of rodents (rats and mice) for experiments on SC related injuries is on the increase, but transgenic and gene-deficient, particularly double- and triple-knockout, mice offer inimitable proposition for focusing studies on specific questions (Myers et al., 2011, 2014; Fassbender et al., 2012).

## Inflammatory Environment Subsequent to Primary Injury (Secondary Injury)

**Figure 2:**
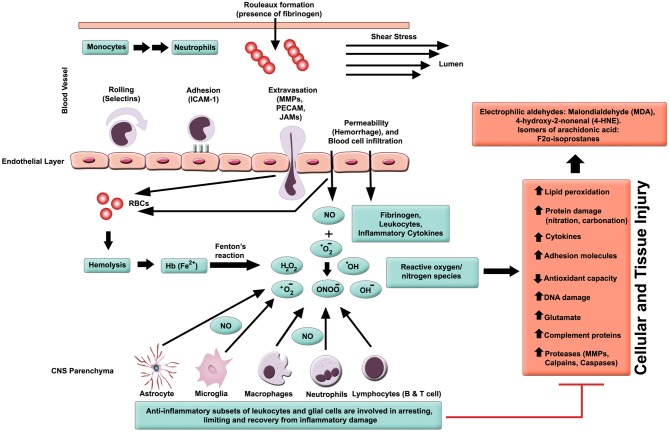
**Inflammatory milieu produced by secondary injury.** An increase in vascular permeability is initiated by hemorrhaging. This is then followed by extravasation of activated leukocytes, which release inflammatory ligands (such as MMPs to degrade extracellular matrix and intercellular proteins). Interaction between ferrous iron (Fe^2+^) and hydrogen peroxide yields hydroxyl radicals (Fenton reaction). Ferric iron (Fe^3+^) reacts with superoxide to produce oxygen (Haber-Weiss reaction). Reactive oxygen species (ROS) activates proteins with cysteine-rich residues by structural modification (oxidation, nitration), and to induce multiple signaling pathways that also modulate gene expression. Intracellular levels of anti-oxidants are depleted in reducing Fe^3+^ to Fe^2+^ state. Ultimately, the free radicals lead to cell and tissue damage, resulting in neuronal and glial necrosis and apoptosis in SC parenchyma. In contrast, beneficial effects are mediated through subsets of leukocytes and glial cells, which play a crucial role in anti-inflammatory mechanisms.

**Figure 3:**
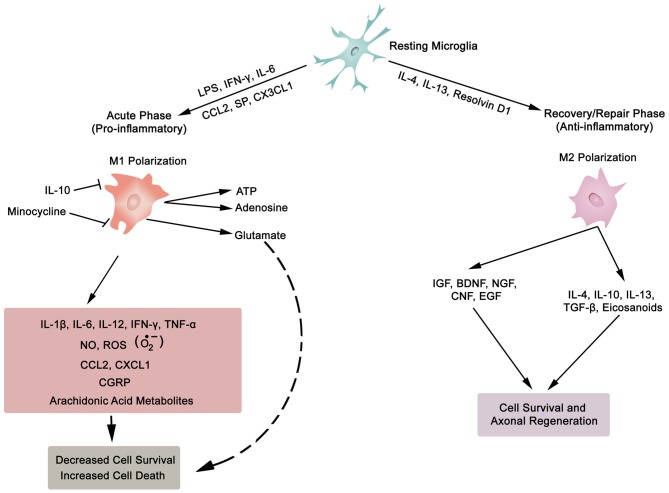
**Synopsis of microglia phenotypes (M1 and M2), the two ends of the biological spectrum.** Inflammatory cytokines (IL-1β, IL-6, IL-12, IFN-γ, TNF-α), anti-inflammatory cytokines (IL-4, IL-10, IL-13, TGF-β), chemokines (CCL2, CXCL1, CX3CL1), growth factors (IGF, insulin-like growth factor; BDNF, brain-derived neurotrophic factor; NGF, nerve growth factor; CNF, ciliary neurotrophic factor; EGF, epidermal growth factor), SP, substance P; NO, nitric oxide; ROS, reactive oxygen species; ${\text{O}}_{2}^{•-}$, superoxide; CGRP, calcitonin gene-related peptide; LPS, lipopolysaccharide; ATP, adenosine triphosphate; Resolvin D1, lipid mediator derived from docosahexaenoic acid. Also, asterocytes display similar phenotypic polarization.

**Figure 4:**
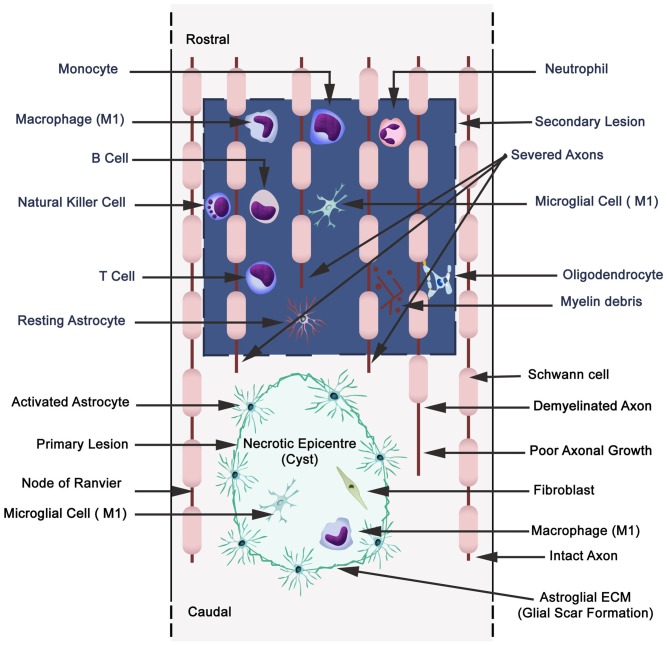
**Schematic illustration displaying primary (cavitation) and secondary lesions, neuronal necrosis, axonal destruction and demyelination during secondary injury with parenchymal resident cells (reactive astrocytes, microglia), and extravasation of peripheral leukocytes (neutrophils, monocytes/macrophages, and lymphocytes: B,T and natural killer cells).** Wallerian degeneration (microtubules disassembly, microtubule associated protein degradation by calcium-dependent neutral protease calpain, blebbing of axons, fragmentation and phagocytosis by microglia and macrophages). A small quantity of Schwann cells are present in CNS, and also they migrate to the CNS from the peripheral nervous system.

**Figure 5:**
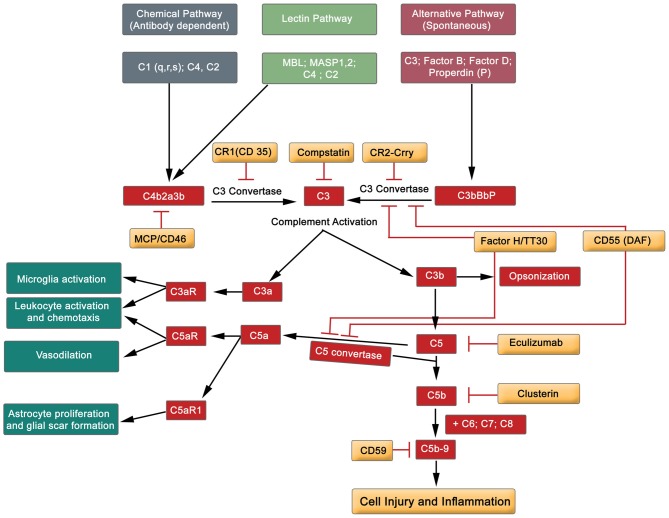
**Simplified diagram exhibiting the major components of the complement cascade, including regulators of complement activation and pathophysiological effects**.

In general, inflammation is the body’s adaptive, homeostatic response to localized-injury, and is instrumental in limiting and repairing the tissue damage if the compensatory pathways are still functional. Otherwise, a decompensatory state follows leading to multi-organ dysregulation and inevitable death (Popovich et al., 1997; Guízar-Sahagún et al., 2004; Medzhitov, 2010; David et al., 2012; Allison and Ditor, 2015). Specifically for SCI, the primary insult inflicted on SC (results in fracture and/or distortion of the SC, damage to axons, blood vessels and neurons, including shearing/stretching of cell membranes) acts as a nexus from which subsequent temporal-profile of secondary damage emanates, which may extend to months and years subsequent to the initial trauma (Table 1, Figures 2, 4). This milieu initiates secondary injury concomitant with inflammatory cascades arising from innate (peripheral: neutrophils, monocytes and macrophages; resident cells of CNS parenchyma: astrocytes and microglia) and adaptive (B and T lymphocytes) immune responses (Figures 2, 3, 4). The array of released inflammogens encapsulates pro-inflammatory cytokines (IFN-γ, TNF-α, IL-1, IL-6, IL-8 and IL-12)/chemokines (CXCL1, CXCL12), nitric oxide (iNOS), oxidants, glutaminergic ions, proteases (matrix metallo-proteinases, calpains, caspases), complement proteins. Ultimately, the outcome from the action of these mediators is necrotic and apoptotic cell death with an impact on functional behavior (autonomic, motor, sensory and reflex), (Table 1, Figures 2, 3, 4, 5; Popovich et al., 1997; Guízar-Sahagún et al., 2004; David et al., 2012; Allison and Ditor, 2015). In parallel, a compensatory anti-inflammatory response stemming from same cells with a different phenotype ensues to repair, be neuroprotective and neuroregenerative (Cherry et al., 2014; Witcher et al., 2015). As such, a comprehension of the principal multi-faceted mechanisms is of paramount importance in facilitating a way to therapeutic strategies. Hence, in this section, we describe some of the key players in disseminating the secondary injury, with a perceptive eye for potential targets for treatment of SCI.

### Hemorrhage

Primary mechanical impact results in immediate localized changes in vascular tissue as well as a reduction in blood supply to the area of SCI (Dumont et al., 2001). These alterations rapidly progress to hemorrhagic and ischemic pathogenesis. The former is primarily located within the gray matter, while the white matter becomes edematous (Tator and Koyanagi, 1997; Mautes et al., 2000; Losey et al., 2014).

Blood infiltrating into the perivascular space in the gray matter has been reported to be noxious to parenchymal cells, eventually leading to neuronal and axonal damage (Asano, 1980). The internal bleeding initiates multiple inflammatory events including but not limited to elevated thrombin formation. This is actually coupled to increased circulating noradrenaline concentrations which eventually lead to activation of platelets and therefore thrombogenesis. It is important to note that upon degradation, components of hemolysates, the lysed products of red blood cells, will discharge iron, which is rather toxic (Dumont et al., 2001; Hua et al., 2006).

### Ischemia-Reperfusion Injury

After the mechanical impact of initial injury to the SC and surrounding tissue, what follows is a prolonged period of secondary injury. This ensues in phases, which range from acute (immediately; minutes) to chronic (years; Table 1). Such injury is primarily caused by hypo-perfused region in the spinal zone resulting in impairment of the SC (epicenter and penumbra). This region becomes susceptible to ischemic damage with breakdown of BSCB. Some hallmarks include edema, hemorrhaging, endothelial permeability and loss of autoregulation. These often involve the participation of multiple components, including different cell types and biochemical pathways. Disruption of blood flow results in local cell injury and infarction caused by hypoxia, ischemia and thrombosis (Fassbender et al., 2011). This pathological trigger initiates a complex, multi-factorial event that is facilitated by inflammatory mediators, cell death (apoptotic, autophagic and necrotic), immune route, oxidative stress and vascular dysfunction (loss of autoregulation). This alteration in homeostatic status not only affects the primary locality of injury but also has an effect on the entire persona of the individual, encompassing somatic as well as psychometric derangement (Maldonado Bouchard and Hook, 2014).

Ischemia reperfusion injury is associated with all of the aforementioned animal models, and is a key player in inducing a robust immune-response in the affected area of cord tissue (Zhu et al., 2013). This is characterized by infiltration of inflammatory cells, such as leukocytes (macrophages, neutrophils and lymphocytes), astrocytes and microglia. These activated cells release inflammatory cytokines and other molecular entities that alter cellular, tissue and organ homeostasis.

### Microglia and Astroglia

#### Microglial Cells

Neurons (excitable) and glia (non-excitable, astrocytes, ependymal, oligodendrocytes and microglia) cells constitute the majority of cellular elements in CNS. The ratio of glia to neurons is almost equal to one (occupying approximately 50% of the volume of CNS; Azevedo et al., 2009), and glial cells collectively serve in playing a homeostatic role to support and protect neurons. Astroglia and microglia choreograph the innate immune responses of the spinal cord (SC) and brain. Following a SC trauma (vertebra fractures, displacement), microglia on sensing cues of cellular or tissue damage (DAMPs, including myelin and cellular debris, IFN-γ, laminin, or ATP) via toll-like receptors (TLRs) in their niche (Olson and Miller, 2004; Lee et al., 2007; Lehnardt, 2010; Heiman et al., 2014), become activated, undergo hypertrophic morphological and functional changes, as well as transform to a migratory mode (Witcher et al., 2015). Traditionally, microglial cells (save for the quiescent state—ramified morphology) exist in two basic polarised states, which are dependent on external signals, the M1 (classical activated) pro-inflammatory phenotype and M2 (alternatively activated) anti-inflammatory phenotype maintain SC homeostasis (Durafourt et al., 2012). The two activated phenotypes (M1 and M2) appear with deramification morphology, being transformed into amoeboid shape (Lee et al., 2007). Activated M1 microglia, initiate cascades of neurotoxic responses in secondary phase of SCI, and significantly contribute to the preponderance of damage (apoptosis and necrosis) to endothelia, neurons, axons and oligodendrocytes, and finally phagocytosis (Blight, 1992; Dusart and Schwab, 1994; Sekhon and Fehlings, 2001; Nguyen et al., 2012). The inflammatory cascades are channeled through multiple pathophysiological signaling pathways, including those of cytokines (TNF-α, IL-1β, IL-6; Rothwell et al., 1997; Pan et al., 2002; Pineau and Lacroix, 2007), chemokines (Mantovani et al., 2004), iNOS (Merrill et al., 1993; Matsui et al., 2010), reactive oxygen species (ROS; Barger et al., 2007), glutamate (Barger et al., 2007), and proteases (Shields et al., 2000), as illustrated in Figure 3. The expression of inflammatory cytokines is evident within two hours from the initial impact of the mechanical injury (Pan et al., 2002; Pineau and Lacroix, 2007; Table 1). Further, activated M1 microglia are characterized by cell surface [such as CD45 (lymphocyte common antigen) and CD11b (complement receptor 3, integrin α_M_β_2_)] and intracellular (inducible nitric oxide synthase 2, NOS-2) markers. In contrast, the anti-inflammatory phenotype M2 is symbolized by the release of anti-inflammatory cytokines (IL-4, Il-10, IL-13), extrinsic (CD206—mannose receptor, CD163—scavenger receptor) and intrinsic (arginase) biomarkers (Witcher et al., 2015; Durafourt et al., 2012; Thompson et al., 2013).

Lipocalin 2 (LCN2), an acute phase protein, displays myriad of biological functions, which also have an impact on the CNS (Ferreira et al., 2015; Jha et al., 2015). LCN2 is known to promote the conversion of microglia in resting state to M1 phenotype. Moreover, the authors of this investigation pointed out that M1 polarized microglia secrete LCN2, which thereby establishes a vicious inflammatory micro-environment (Jang et al., 2013). In addition, LCN2 not only suppresses the polarization to M2 phenotype by antagonizing the phosphorylation of STAT 6 in IL-4 stimulated microglia, but also sensitizes microglia to auto-death (Lee et al., 2007). On the other hand, IL-10 polarizes microglia to the protective M2 phenotype (Thompson et al., 2013). To sum, microglia, with distinct phenotypes (M1 or M2), regulate the development and maintenance of inflammatory response in SC. This is biologically manifested through the release of a complex and diverse molecular arsenal (detrimental or beneficial) for neurodegenerative, neuro-protective and neuro-reparative events.

Polarization into M1 and M2 microglia phenotypes are the two extreme ends of the spectrum. In between, there is a continuum of states with cells being defined by unique molecular signatures (Cherry et al., 2014). Each of these cellular sub-states takes its cue from the changing micro-milieu, and responds accordingly by adapting their functional phenotype to directly target the emerging challenge. However, a recent study has implied that once the insult is contained, the microglia subsequently become primed for further stresses with a more aggressive immuno-inflammatory response (Witcher et al., 2015). It is feasible that these primed microglia are probably programmed via epigenetic mechanisms, which are known to influence various pathologies, such as hypertension (Al Disi et al., 2015; Anwar et al., 2016a,b), and CNS-related disorders (Sng and Meaney, 2009; Lubin et al., 2011; Tang et al., 2014). Indeed, epigenetic mechanisms are recognized to program different SC cell populations (glial, immune and neuronal; York et al., 2013). Taken together, this reflects the diversity and functional plasticity, including the immunoregulatory roles of microglia in SCI. In order to make use of microglia diversity, a profound comprehension of regulatory mechanisms is required for optimum therapeutic strategy, particularly in relation to neurorepair and neuroregeneration of SC.

#### Astrocytes

Astrocytes comprise of a heterogeneous population of cells with diverse functional capacity (Tabata, 2015). An essential mechanistic operation of these glia cells is their significant role in maintaining the homeostasis of CNS (Perea and Araque, 2007; Paixão and Klein, 2010). In contrast, the astroglia, a component of the innate immunity system, are considered to be involved in noxious developments of secondary damage (Table 1). Recent analyses have revealed the diversity and plasticity that exists in astroglia reflected by identification of functional polarization states (classically activated M1 type [synthesize proinflammatory cytokines, growth factors, NO, glutamate and reactive oxygen and nitrogen species] and alternatively activated M2 type [produce anti-inflammatory releasates]) which are dependent on the extracellular environment for activation. Apparently, the two states are similar to macrophages and microglial cells (Cherry et al., 2014), and presumably there may be an array of intermediate states (Cherry et al., 2014).

Extravasated neutrophils secrete proteolytic enzymes causing injury to neurons, glia, and endothelial cells (Schwab and Bartholdi, 1996). Subsequently, macrophages and microglia are recruited, which not only phagocytose cellular and molecular debris but contribute to demyelination of axons, apoptosis of neurons and oligodendrocytes after the initial insult (Merrill et al., 1993; Shuman et al., 1997; McTigue and Tripathi, 2008; Hall and Traystman, 2009). At this stage, regions of cavitation are apparent, and Wallerian degradation with associated glial scarring (prominence of astrocytes) is notable (Schwab and Bartholdi, 1996). Inflammatory environment prompts astrocytes (hypertrophic, migratory and proliferative) to ring fence the injured area by formation of a glial scar, which forms a barrier to regrowth of axons. Interestingly, both microglia (astrocytes and oligodendrocytes) express inhibitory factors (chondroitin sulfate proteoglycans, myelin associated glycoprotein, Nogo-A, oligodendrocyte-myelin glycoprotein and tenascin) to block axonal regeneration. These are potential targets for therapeutic manipulation.

It has been promulgated that in both microglia and astroglia antioxidant exhaustion (glutathione) is induced by hyperactivity of NADPH oxidase, which elicits excessive secretion of neurotoxicant glutamate with associated neuronal and oligodendrocyte loss (Barger et al., 2007; Johnstone et al., 2013). Activation of NMDA receptors by glutamate triggers influx of calcium, which is a cofactor activating calpain (potently degrades axon/myelin), a neutral cysteine protease (Shields et al., 2000). Therefore, therapeutically targeting oxidative enzymes will prevent axonal demyelination, preserve oligodendrocyte integrity and spare neuronal loss (Johnstone et al., 2013).

Astrocytes contribute to progression of axonal degradation, neuronal death and motor/sensory functional deficit following SCI. Several options are available to therapeutically target astroglia, such as development of agents that reduce the density of polarised M1 astroglia in SC, and to increase the anti-inflammatory M2 type astroglia.

### Oxidative Stress

In able-bodied individuals, the activity of oxidative species (free radicals and ROS) is balanced by cellular anti-oxidant agents. Chronic levels of ROS accompany many inflammatory diseases, including hypertension, atherosclerosis, neurodegeneration, IRI and cancer (D’Autreaux and Toledano, 2007; Yang et al., 2013). Concerning SCI subjects, a milieu is created that drives a shift in balance towards oxidative stress (pro-oxidant state), and along with other cues, this evolves into an inflammatory pathology (Bains and Hall, 2012). The increase in oxidative stress is also correlated with progression of age, but there is a reciprocal decline in concentrations of antioxidants. Consequently, the severity of SCI rises with age and is dependent on pro-oxidant/anti-oxidant balance (Shao et al., 2006). Activated parenchyma (microglia) and leukocytes (macrophages and neutrophils) are the main sources of ROS (Bains and Hall, 2012; Figure 2). The principal components of ROS are the superoxides (${\text{O}}_{2}^{•-}$), hydroxyl radicals (•OH, a product of Fenton reaction), hydrogen peroxides (H_2_O_2_), nitric oxide (•NO) and peroxynitrites (ONOO−) amongst others (Xiong and Hall, 2009; Yang et al., 2013). These are synthesized by enzyme systems (nicotinamide-adenine dinucleotide phosphate (NADPH) oxidase, myeloperoxidases, cyclooxygenase, and xanthine oxidase), both in microglia and leukocytes (D’Autreaux and Toledano, 2007; Bains and Hall, 2012).

Detrimental effects of oxidative stress pathways commence immediately after the primary impact of SCI, causing membrane and cellular damage (Silva et al., 2014). Apparently, the superoxide ${\text{O}}_{2}^{•-}$ (Liu et al., 1998) and •OH (Bao and Liu, 2004) are the principle culprits in contused SCI.

Polyunsaturated fatty acids (arachidonic acid, docosahexaenoic acid) are targets for free radicals, producing highly reactive electrophilic aldehydes, such as malondialdehyde (MDA; (Qian and Liu, 1997), 4-hydroxy-2-nonenal (4-HNE; (Baldwin et al., 1998) and acrolein (Luo et al., 2005), all of which are considered as biomarkers of oxidative injury (Figure 2). Enzymatic (cyclooxygenase) and non-enzymatic oxidation of arachidonic acid also yield 8-iso-prostaglandin F_2α_, which again is a marker of lipid peroxidation (Clausen et al., 2012). The reactive aldehydes damage the blood spinal cord barrier (BSCB; Mullick et al., 2002; Ellis, 2007), causing decrease in cell viability (Ayala et al., 2014) and hence an increase in vascular permeability (Huber et al., 2002). Conversely, the lipid peroxidation end products are inactivated by aldehyde dehydrogenases and other enzymes such as aldehyde reductases, glutathione S-transferases (Ellis, 2007; Ayala et al., 2014).

The oxidant reactants are inactivated by intra- and extra-cellular antioxidant defense systems like the enzymatic superoxide dismutases (SOD), catalase, glutathione peroxidase, glutathione reductase, and non-enzymatic antioxidants (vitamins A, E and C; glutathione; carotenoids and flavonoids; Bains and Hall, 2012). It has been stressed that the therapeutic window is time-dependent in SCI, and should be triggered as early as possible (<3 h), not only to curtail the pathology, but also to quench the oxidative reactants (Bains and Hall, 2012). Consequently, anti-oxidative therapy may arrest and reverse the inflammatory response in SCI.

### Nitric Oxide

Nitric oxide (NO) participates in pleiotropic activities as a mediator of physiological and pathophysiological processes including immunoregulation (Moncada et al., 1991; Toda et al., 2009). It is synthesized from arginine by NOS, which exists in three different isoforms: neuronal (nNOS, NOS-2), the inducible form (iNOS, NOS-2), and the endothelial enzyme (eNOS, NOS-3). These isoforms are expressed and located in a variety of cell types and tissues (Toda et al., 2009; Sheng et al., 2011).

Activated eNOS releases the vasodilating NO, which maintains vascular homeostatic signaling by modulating arterial tone, and hence regulating blood pressure. However, when NO production is impaired, endothelial cell dysfunction ensues, leading to cardio- and cerebrovascular diseases (Moncada et al., 1991; Toda et al., 2009).

Inflammatory cytokines (TNF-α, IFN and IL-1) and glycosphingolipids are recognized for their induction of iNOS in a broad spectrum of cell types, including astrocytes, microglia, macrophages, and neurons (Satake et al., 2000; Beattie, 2004; Toda et al., 2009; Sheng et al., 2011). The quantity of NO generated by iNOS is normally far in excess of that produced by other isoforms, and iNOS is highly implicated in inflammatory processes such as SCI (Conti et al., 2007; Maggio et al., 2012). iNOS produces excessive amounts of NO molecules which react with superoxide radicals to generate reactive nitrogen species (Pannu and Singh, 2006). Activation of such oxidative microcosm triggers lipid peroxidation, DNA fragmentation and blockade of mitochondrial respiration. Hence, accumulation of these pathological pathways leads to the degeneration of resident cells of parenchyma (Maggio et al., 2012). NO has been implicated in microglia-dependent demyelination and apoptosis of neuronal cells (Pannu and Singh, 2006). The situation with the use of inhibitors for NOS isoforms is complex. Studies with iNOS blockers (1400W and aminoguanidine) have resulted in further neuronal/oligodendrocyte deterioration instead of neurological recovery (Pannu and Singh, 2006). Additional studies into expression, activity, temporal and spatial distribution of NOS family of enzymes at the site of SCI are required, particularly in relation to NO-derived secondary damage. This is to ultimately achieve an optimum level of NO for physiological homeostasis and therapeutically targeting the enzymes (Marsala et al., 2007; Tardivo et al., 2015).

### Fibrinogen

Fibrinogen is an acute phase protein, a member of the clotting cascade, an orchestrator of other diverse functional activities, and a biomarker for excess risk of cardiovascular disease (Kamath and Lip, 2003; Mosesson, 2005; Cray et al., 2009; Jennewein et al., 2011). Evidence shows it may also play a significant pro-inflammatory role in secondary injury following primary SCI (Wu et al., 1994). Coagulation abnormalities, particularly with modest elevation in plasma fibrinogen concentration, are associated with SCI (Pahl et al., 1994). Immediately following SCI, thrombotic activity is a major clinical problem, which subsides over time with recruitment of the fibrinolytic pathway (Frisbie, 2006). A clear illustration of fibrinogen’s role in post-SCI inflammatory process is highlighted by its activation of microglia cells (Davalos et al., 2012), which are notoriously pro-inflammatory participants in secondary injury (Fleming et al., 2006; Zhou et al., 2014). Fibrinogen-triggered astrocyte-stimulation directs scar formation (Schachtrup et al., 2010), and it also plays a role in activation of microglial cells by binding to α_M_β_2_ integrins (Jennewein et al., 2011). As such, fibrinogen has an important function to do by modulating the activity of parenchymal cells. Moreover, fibrinogen promotes homotypic and heterotypic cellular adhesion and aggregation. Indeed, increasing concentration of fibrinogen correlates with elevated red cell aggregation and formation of a rouleaux type structures (Rampling, 1981; Rampling and Challoner, 1983; Anwar et al., 1994). These structures retard the circulatory flow, especially in low flow areas of pre-capillary arterioles and post-capillary venules (McHedlishvili et al., 1999; Pearson and Lipowsky, 2004). This scenario causes low-grade inflammation, a pro-coagulant state with localized endothelial dysfunction (Zilliacus, 1951; Gavins et al., 2011; Jennewein et al., 2011). Further, fibrinogen is a potent aggregator of white blood cells and platelets (Kamath and Lip, 2003), the latter being reported to have impaired function in SCI individuals, placing these patients at an increased risk for coronary heart disease (athero-thrombogenesis; Khan et al., 2011). Furthermore, fibrinogen binds to stimulated leukocytes (Jennewein et al., 2011), which initially roll along the lumen wall, then adhere to endothelial cells, followed by extravasation (trans-endothelial migration) to the focal area of injury where they exert their inflammatory and anti-inflammatory functions (Belch et al., 1998; Kirschenbaum et al., 2004; Lominadze et al., 2010; Jennewein et al., 2011; Figure 2). Taken together, it becomes apparent that fibrinogen plays a critical role in SCI pathology, embracing inflammation, coagulation, hemostasis and cellular interactions.

### Leukocytes

Leukocytes (granulocytes, monocytes/macrophages and lymphocytes) are recruited to the epicenter of SCI, where they not only participate in inflammatory SCI-induced events, but are also involved in limiting and repairing of the pathological damage, hence imparting an anti-inflammatory effect *per se* (Figure 4; Fleming et al., 2006; Zhang and Gensel, 2014). Evidently, there is mounting recognition for the existence in heterogeneity related to neuro-inflammation between brain and spinal cord following an identical mechanical trauma. This is in terms of a more robust response from activated granulocytes, mononuclear (B and T cells) and microglia cells in SCI compared to the brain (Batchelor et al., 2008; Zhang and Gensel, 2014). Neutrophils are the first inflammatory cells to migrate to the site of injury with an arsenal of oxidative (NADP oxidase and myeloperoxidase) and proteolytic enzymes (matrix metalloproteinase-9; MMP-9; Fleming et al., 2006; Kolaczkowska and Kubes, 2013). Hence, inhibiting the recruitment of neutrophils and monocytes has been reported to be beneficial in SCI environment (Lee et al., 2011). In contrast, a recent report implied that subtypes of neutrophils encourage recovery from inflammatory pathology during the secondary phase of SCI following the catastrophic insult of mechanical forces in primary SCI (Neirinckx et al., 2014).

Macrophages are divided into two main subtypes, which upon activation release bioactive mediators like cytokines, nitric oxide and eicosanoids. The role of pro- (M1) and anti-inflammatory (M2) macrophages in SCI is slowly being deciphered in detail (Zhang and Gensel, 2014). Particular attention is being paid in relation to the ratio of pro-inflammatory macrophages (cytokines: IL-1β, IL-6, IL-12, TNF-α) to that of anti-inflammatory type (IL-10, IL-13) during ischemia and in resolution of the inflammatory response (Shechter et al., 2009; Wynn et al., 2013; Brown et al., 2014; Zhou et al., 2014). It is worth mentioning that these macrophages also have a role in wound healing. Contextually, there is further subdivision of M1 and M2 (M2a—involved in wound healing, M2b and M2c) macrophage phenotypes (Parsa et al., 2012). Further, these investigators alluded to the importance of macrophage diversity in terms of individual’s immune susceptibility to inflammation and resolution (Parsa et al., 2012).

The density of lymphocytes (B and T) in tissue at the location of SCI is considerably lower than other leukocytes (Bradl et al., 2005). Once again, there is heterogeneity in distribution of CD4^+^ and CD8^+^ T cells, with CD4^+^ subset being quantitatively greater than CD8^+^ lymphocytes in regions of degenerative rat SC (Bradl et al., 2005). However, B-lymphocytes are known to exert complex pathophysiological effects, including facilitating anti-inflammatory and/or pro-inflammatory actions. In SCI, elevated concentrations of two cytokines (B-cell-activating factor, BAFF, and a proliferation-inducing ligand, APRIL) and B-cell maturation antigen (BCMA) have been associated with B cells. The latter activates B-cells and the cytokines regulate SCI-autoimmunity (Saltzman et al., 2013). Hence, these proteins are plausible targets for neuro-immune management. Hopefully, in the next few years these dichotomous effects of leukocytes will be clarified in detail, with further emphasis on pro- and anti-inflammatory secretagogue fingerprints, which can then be targeted for therapeutically beneficial effects.

### The Complement System

Complement factors, part of the innate immune system, are emerging as significant players in inflammatory neuropathogenesis of SCI (Alexander et al., 2008; Peterson and Anderson, 2014). In contrast, complement is also associated with neuroprotection, repair and regeneration (Alexander et al., 2008; Alawieh et al., 2015). Hence, the proteins display a Janus face of dual activity, beneficial on one hand and detrimental on the reverse side. Similarly, complement activity is increased in experimental models (Galvan et al., 2008), and in sera of patients with SCI (Rebhun et al., 1991). Further, the factors also contribute to post-reperfusion recovery (Alawieh et al., 2015). Therefore, the aforementioned have aroused interest in the use of complement inhibitors as therapeutic agents in the SCI field.

The complement system comprises of almost 60 protein members, including activators and inhibitors (Liszewski and Atkinson, 2015). Briefly, the complement system can be activated by three different pathways: classical, lectin and alternative routes (Figure 5). These three pathways initially converge at C3, and finally at terminal position by the formation of C5b-9, the cytolytic membrane attack complex (MAC). Complement proteins C3a and C5a participate in both, depending on the context, proinflammatory and neuroprotective role (Figure 5; Woodruff et al., 2010; Merle et al., 2015). Hence, the activation of the complement cascade results in amplification of the inflammatory response by many-fold through the recruitment and activation of the immune system (adaptive and innate, see Table 1). Endogenously produced cell membrane-anchored inhibitors protect against complement activation, thereby playing a role in homeostatic functions. This diverse group embraces the complement receptor 1 (CR1, and Crry—a rodent homolog of human CR1), decay-accelerating factor (DAF, CD55) and CD59 (glycosylphosphtidyl protein), all of which are recognized to play a regulatory role in complement activation (Figure 5; Merle et al., 2015). Further, the serum factor H modulates the proteolytic activity at different points of the complement cascade, and is the principle regulator of complement protein C3b (Jokiranta et al., 2000; Wu et al., 2009). Another set of complement factor H-related proteins, originally considered to be antagonists, but recent studies have redefined them as activators of complement proteins (Józsi et al., 2015; Figure 5). Rodents treated with endogenous inhibitors and complement gene-deficient mice (factor B, C1q and C3) have demonstrated neuronal protection subsequent to SCI, and improved sensory and locomotor functions (Galvan et al., 2008; Brennan et al., 2012; Peterson et al., 2015).

Accumulated evidence gives credence to the safety and efficacy of complement protein directed therapeutic agents in equilibrating this overactive innate immune system. This is reflected by the application of diverse range of novel inhibitors directed at different protein constituents of the complement cascade (Figure 5). Namely, Eculizumab (a monoclonal antibody targeting protein C5) has been used to treat paroxysmal nocturnal hemoglobinuria (PNH) patients. Thus, Eculizumab antagonizes the formation of C5b-9 (MAC), and this effectively reduces hemolysis in the patients (Rother et al., 2007; Brodsky et al., 2008). Another protein that has recently been tested on erythrocytes is TT30 (C3d-targeted C3/C5 convertase inhibitor (Fridkis-Hareli et al., 2011; Risitano et al., 2012), which prevents complement-mediated lysis of erythrocytes. More recently, a small molecule anti-complement agent, Compstatin (a peptide inhibitor of central complement C3) is generating enormous amount of excitement in clinical environment to eliminate complement related diseases (Mastellos et al., 2015). Compstatin is recognized to block complement activation of RBCs of PNH patients (Janssen et al., 2007; Risitano et al., 2014). To date, these tested, safe and efficacious inhibitors have not been applied to alleviate the impact of traumatic SCI on patients. The horizon is bright with therapeutic reagents.

### Apoptosis

Necrotogenic and apoptogenic mechanisms determine the dimensions of SC lesion during the time-frame of secondary injury that follows the primary physical impact of SCI (Beattie et al., 2000). Apoptosis occurs in the parenchymal cells of SC that include astrocytes, microglia, oligodendrocytes and neurons (Beattie et al., 2000). Activation of microglia cells is related to oligodendrocyte cell death and axon demyelination, which are all attenuated with application of minocycline, an antibiotic (Beattie et al., 2000). Indeed, a concomitant recovery in neuro-function is reported with this treatment (Beattie et al., 2000). Therefore, early application of anti-apoptotic therapy will be a step in direction to recovery from secondary injury.

## Inflammatory Effects on Circulation

### Effects on SC Vascular Bed

Continuing from the aforementioned (“Animal Models for SCI” Section), the inflammatory cascade has an immediate effect on SC tissue and the peripheral circulation. Subjects with SCI are at an increased risk of cardiovascular disturbances that contribute to approximately 40% of deaths in these individuals (Garshick et al., 2005). In addition, in a significant proportion of SCI individuals, hypertension, an inflammatory condition, is a prevalent cardiovascular risk factor (Lee et al., 2006; Selassie et al., 2013).

The splanchnic and the skeletal muscle vascular beds account for over 50% total blood pool, and in case of emergency this pool of blood is diverted to the cerebral, cardiac and adrenal circulations. Blood is transported to the SC through branches arising from the aorta. The vascular tree feeding the SC is complex and extensive in nature along the length of the cord (Bosmia et al., 2015). It includes three arteries that cater for the blood supply to the SC: the anterior spinal artery (conduit for over 70% of blood supply to the SC; and a branch of vertebral artery arising from the subclavian artery) and the paired posterior spinal arteries (Bosmia et al., 2015). The microcirculation in the cord consists of the sulcal (anterior) and pial (anterior, in combination with branches of posterior arteries) arterial networks. However, the microvascular system (centrifugal) delivers blood to a large portion of gray matter (mostly neurons) and inner half of the white matter (mostly myelinated tracts). Furthermore, the pial microarterial bed, in conjunction with outlets from branches of posterior arteries, provides blood to the posterior white matter and dorsal horns (Losey and Anthony, 2014). Interestingly, the gray matter is highly vascularized compared to white matter, which has a lower metabolic rate due to low arterial density (Martirosyan et al., 2011).

Autoregulation is a notably important and well-developed regulatory mechanism in the vascular system, especially in response to high levels of carbon dioxide. However, during SCI, the autoregulation of hemodynamics in the SC vasculature becomes dysregulated (Bosmia et al., 2015). In contrast to the cerebral circulation, there is paucity of data on the pharmacological response of arteries to vasoactive agents from different locations of the SC, particularly isolated vessels that would remove any *in situ* confounding elements (Tator and Koyanagi, 1997; Bosmia et al., 2015). In this regard, further studies are warranted to comprehend how different regions of the SC respond in health and trauma (inflammation), and this would potentially help in therapeutically maintaining blood supply to the injured SC.

Vascular endothelial lining controls physiological properties, and hence it is not surprising that endothelial impairment promotes SC injuries (Popa et al., 2010; Fassbender et al., 2011). Indeed, activation of endothelial NO synthase (eNOS) releases NO, which regulates vascular tone (Toda et al., 2009). However, cardiovascular derangement ensues if the activity/expression of eNOS pathway is impaired. This happens to be the most common signaling route for cardiovascular dysfunction throughout the vascular network, including SCI (West et al., 2013). Endothelial function is also dependent on endothelial-derived hyperpolarizing factor, EDHF (Gerber et al., 1998; Félétou and Vanhoutte, 2006), but to our knowledge no study has been conducted on this important molecular transducer in the SCI condition. In the context of inflammogenesis, pro-inflammatory mediators dampen the generation of EDHF (Kessler et al., 1999). Further, inflammatory cytokines like TNF-α activate NADPH oxidase to trigger the release of ROS, which induces endothelial dysfunction (Gao et al., 2007; Zhang et al., 2009).

Degree of severity in the inflammatory pathology of SCI is a distinct determinant of cardiovascular outcome, not only in the SC vascular bed (Martirosyan et al., 2011; Sharma, 2011), but also the peripheral circulation (West et al., 2013). Abnormalities associated with SC circulation have been implicated in stroke, which is an inflammatory condition as well (Popa et al., 2010; Sharma, 2011). Therefore, therapeutic strategies aimed at the early stage of inflammatory activation in the microcirculatory network of SCI patients are pivotal to neuroprotection (Sharma, 2011).

### Effects on Peripheral Circulatory Network

An alteration in vascular function, particularly below the cord lesion, is a key event in inflammogenesis of secondary injury. This includes ischemic injury that sets in motion an array of pathological pathways. Inflammatory biodegradation of the cord-blood barrier further exacerbates the pathology of secondary injury (hemorrhaging, vascular permeability and edema). Moreover, the systemic, specifically peripheral, circulation is profoundly affected (West et al., 2013). Interestingly, the dysfunction in vascular reactivity observed in lower limbs, reflected by decreased flow mediated dilatation, is greater compared to the upper limbs of SCI patients (Stoner et al., 2006). Similarly, it has been suggested that compromised blood flow in dermal arterioles results in skin lesions, a further indicator of altered blood flow in SCI individuals (Deitrick et al., 2007). Contextually, the normalized intima-media thickness of lower-extremity arteries (superficial femoral and popliteal arteries, structural inward remodeling) is greater in SCI individuals than a healthy able-bodied control group PAD (Bell et al., 2011). This is indicative of subclinical prevalence of lower limb peripheral arterial disease, PAD (Bell et al., 2011). As a matter of fact, PAD is partially driven by an altered lipid profile in these subjects (Bauman and Spungen, 1994). Interestingly, investigators have reported a greater incidence of lower limb PAD associated with higher burden of stroke (Criqui et al., 1992). The afore-mentioned offers an explanation for the observation that patients with SCI are at an increased risk of future ischemic stroke (Wu et al., 2012). Therefore, examining double insults in rats, such as an initial SCI event followed by middle cerebral artery occlusion (MCAO) procedure may be considered a suitable model for a prospective stroke development. Further, thoracic SC sectioning in rats is associated with decrease in microvascular blood flow in several organs, including the liver, spleen and muscle. Apparently, these changes correlate with increase in peripheral arterial resistances, and hence a cause for future organ failure (Guízar-Sahagún et al., 2004).

In conjunction with SCI, the circulatory disturbances may be even more pronounced in the prior presence of established risk factors, such as diabetes, obesity, dyslipidemia, tobacco abuse, alcohol, low self-appreciation or economic well-being, malnutrition, and hypertension. All of which are associated with an inflammatory pathology, where in most cases the microvascular network is already compromised (Granger et al., 2010). Evidently, to our knowledge, no studies have taken place to address these plausible factors in pathophysiology of SCI. However, it has been suggested that patients with SCI demonstrate a distinct physiological profile relative to healthy, able-bodied controls (Wilt et al., 2008). Apparently, an adverse serum lipid profile is a common problem in SCI patients compared to ambulatory controls (Gilbert et al., 2014; Lieberman et al., 2014). This is specifically related to low levels of high density lipoprotein cholesterol, HDL-C (Gilbert et al., 2014; Lieberman et al., 2014). The aforementioned investigations suggest that high quality intervention studies incorporating prior risk factors for CVD must be forthcoming to comprehend and build up an overall picture for SCI individuals.

### Inflammatory Role of Renin/Angiotensin System in SCI

The balance between angiotensin converting enzyme (ACE) and ACE2 of renin angiotensin system (RAS) pathways is of considerable importance for homeostasis in the whole body. The sustained shift in equilibrium towards ACE results in a pathologic state, which often arises in SCI (Groothuis et al., 2010; Popa et al., 2010; Al Dera and Brock, 2015). This leads to stimulation of a network of transducing pathways, including an increase in Ang II-induced vasoconstriction, Ang II-stimulated oxidative stress, as well as cellular proliferation. All of these mechanisms contribute to Ang II-derived inflammation, which in turn participates in secondary injury (Groothuis et al., 2010; Vajapey et al., 2014; Al Dera and Brock, 2015). However, the ACE2/Ang 1-7/Mas route is considered to be neuroprotective (Bennion et al., 2015). At any rate, further studies are needed to better determine the role of RAS in the inflammogenesis of SCI.

### Effects on Rheological Parameters

Hemodynamic factors are important participants in various pathologies including secondary SCI. The determinants of whole blood viscosity include plasma viscosity, erythrocyte and leukocyte aggregation (rouleaux), and red and white blood cell deformability (Hochmuth, 1993; Anwar et al., 1994; Adams and Nash, 1996; Rampling et al., 2004). Plasma viscosity and red cell aggregation are a function of plasma proteins (large, asymmetric), particularly fibrinogen, immunoglobulins (IgM, IgA and IgG), alpha-2-macroglobulin and lipoproteins (Anwar and Rampling, 2003). As indicated earlier, inflammation progressively increases the activation of white blood cells, and decreases white blood cell deformability. Consequentially, the transit time through the circulatory network is significantly increased when an activated, and hence less deformable leukocyte (8 μm, nucleated), which is more than a 1000 times less flexible than an erythrocyte (anucleated), circumnavigates through the capillary network, particularly vessels of dimension 3 μm in diameter (Hochmuth, 1993; Adams and Nash, 1996; Anwar et al., 1999). Leukocytes alter hemodynamics by plugging capillaries in the microcirculation (Adams and Nash, 1996; Anwar et al., 1999).

In a rabbit model of SC ischemia reperfusion injury, a number of observed determinants of hemo-rheological abnormalities, including whole blood viscosity, plasma viscosity, red cell aggregation are increased, whereas erythrocyte deformability is reduced (Zhang et al., 2012). Treatment with Tanshinone-II A sulfonate, a derivative of Salvia miltiorrhiza, of IRI improved all the rheological parameters (Zhang et al., 2012). Apart from this single study (Zhang et al., 2012), there is a clear lack of data with regards to hemo-rheological properties in SCI injury. This is surprising as there is a strong relationship between inflammatory processes and hemo-rheological parameters (Rampling, 1998). Indeed, inflammation drives leukocyte stimulation (becoming adhesive, poorly deformable, releasing proteolytic enzymes) and activates the clotting cascade. Together, these changes lead to fibrinogen heterogeneity, and hence alteration in rheological properties of blood (Rampling, 1998). Moreover, viscometric resistance is a contributory factor to total peripheral resistance that is a significant variable affecting blood pressure. Both hemo-rheological and vascular complications may, therefore, be implicated in observations regarding blood pressure oscillations in earlier and later stages of SCI (West et al., 2013).

## Novel Therapeutic Strategies for Ameliorating SCI Burden

Methylprednisolone (MP), a synthetic glucocorticoid, remains the mainstay for treatment of SCI. This stems mainly from the anti-inflammatory nature of the drug and protection from peroxidation of membrane lipids (Bracken, 2001). But, the corticosteroid suffers from a major short-coming in that of neurological competence, such as lack of neurite sprouting and remyelination of spared axons, and hence a deficit in functional recovery (Constantini and Young, 1994; Ito et al., 2009). In addition, the associated detrimental side-effects of the steroid (gastrointestinal bleeding, pulmonary complications, and sepsis), particularly at high doses of MP, outweigh its primary beneficial property (Ito et al., 2009). From the preceding sections, it becomes evident that in the adult primate CNS, the mature neurons and oligodendrocytes are terminally differentiated as well as inherently devoid of proliferative processes due to inability to produce growth-promoting chemicals (Rakic, 1985; Bhardwaj et al., 2006; Nowakowski, 2006; McTigue and Tripathi, 2008); and the capacity to divide is limited to progenitor/stem cells (Gonzalez-Perez and Alvarez-Buylla, 2011; El Waly et al., 2014). Consequently, for a therapeutic strategy to be potentially effective in alleviating SCI (inflammatory neuro-degeneration), it must demonstrate multiple capacity to be anti-inflammatory, neuro-protective, and neuro-restorative. In this section, we have incorporated some of the treatment approaches that have yielded encouraging results from the lab with potential for translation to the bedside. Moreover, the same modalities are provided in a tabular form to illustrate their application in clinical trials (Table 2).

**Table 2:** **An outline of therapeutic modalities (cell-based, exercise and pharmacological agents), their biological effects, and clinical trials for the treatment of SCI**.

Therapeutic modulator	Biological actions	Status	Study title	Clinicaltrials.gov Identifier
Exercise	Increases skeletal muscle mass as well as cellular, biochemical, and cardiovascular functions; Improves neuroprotection, regeneration and rehabilitative processes	Currently recruiting participants	Study about acting of adaptive sport in musculoskeletal, cardiovascular system and the quality of life of individuals with spinal cord injury through biomedical instrumentation	NCT02177929
Minocycline	Neuroprotective, functional recovery, tissue sparing, down-regulation of pro-inflammatory species	Recruiting	Phase III study of minocycline in acute spinal cord injury	NCT01828203
Cethrin (BA-210)	Inhibitor of Rho/ROCK signaling; reduced apoptosis; decreased glial scarring; regenerative growth of axons	Completed	A safety study for cethrin (BA-210) in the treatment of acute thoracic and cervical spinal	NCT00500812
Erythropoietin	Anti-apoptogenic; anti-inflammatory; improves vascular integrity	Suspended participant recruitment	Evaluation of the tolerability and efficacy of erythropoietin (EPO) treatment in spinal shock: comparative study vs. methylprednisolone (MP)	NCT00561067
Riluzole	Blocks [Na^+^] influx; inhibits glutamatergic neurotransmission; and improves neurological outcome	Currently recruiting participants	Riluzole in spinal cord injury study (RISCIS)	NCT01597518
Hypothermia	Reduces anti-inflammatory species; decreases microglia activation; suppresses neurotoxicity and mitigates blood spinal cord barrier disruption; Anti-apoptogenic	Currently recruiting participants	Hypothermia following acute spinal cord injury	NCT01739010
Cellular approach: *macrophages*	Phagocytosis of cell debris; regeneration of axons; and neurological benefits	Suspended participant recruitment	A Phase II multicenter, randomized- controlled study to evaluate the safety and efficacy of autologous incubated macrophages for the treatment of patients with complete spinal cord injuries	NCT00073853
Cellular approach: bone marrow derived *mesenchymal stem cells*	Promote neuronal regeneration; provide neuroprotection; replace neurons; and neurotrophic factors	Completed	Cell transplant in spinal cord injury Patients	NCT00816803

### Exercise

Following the mechanical impact in causing primary SCI, the secondary damage is responsible for activation of diverse pathophysiological effects and one of the most profound outcomes is the loss in behavioral function, specifically the inability to ambulate. A distinct advantage of exercise is not only that of non-invasive therapy, but as an elixir of well-being, both physical and in terms of psyche of the individual. Exercise imparts positive effects on muscle mass, reduces osteoporosis, and improves cardiovascular endurance and quality of life. Also, it improves the physiology and mood of patients with SC dysfunction through neuroprotective means, neuroregenerative processes and rehabilitative methods, all leading to functional recovery (Sandrow-Feinberg and Houlé, 2015). In a rat model of contusion injury followed by treadmill exercise (duration period of 6 weeks) resulted in suppression of apoptosis (ratio of B-cell lymphoma (Bcl-2) to Bax, and reduced caspase-3 expression). Concomitantly, the activation of PI3K/Akt survival pathway raised the expression of neurotrophic factors (neurotrophin-3, nerve growth factor and insulin-like growth factor). Together, these effects contributed to recovery of motor function (Jung et al., 2014). Exercise is advocated as one of the foremost rehabilitative therapies for SCI, particularly in promoting locomotor capacity; and this has paved the way for conducting clinical trials (Table 2).

### Minocycline

Minocycline (tetracycline antibiotic analog) suppresses microglial activation, and thereby attenuates neuroinflammation in rat contusion injury. In addition, reduced apoptogenesis of oligodendrocytes was associated with suppression of pro-nerve growth factor synthesis in microglia, both *in vivo* and *in vitro*, as a consequence of antagonism of p38 mitogen-activated protein kinase activation. Also, in the same study minocycline blocked the expression of p75 neurotrophin receptor as well as RhoA activation. Collectively, an improvement in functional recovery was noted (Yune et al., 2007). In addition, administration of minocycline to experimental models of SCI led to an elevation in levels of IL-10, which is characterized by anti-inflammatory properties (Thompson et al., 2013). Similarly, minocycline by inhibiting microglial activation and proliferation not only improves the integrity and viability of BSCB (Festoff et al., 2006; Yenari et al., 2006), but also reduces neuronal and glial apoptosis, leading to functional outcome improvements following SCI in experimental models and preclinical studies (Stirling et al., 2004; Festoff et al., 2006; Casha et al., 2012). The biological improvements implied by investigations on minocycline have been stepped-up to clinical trials status (Table 2).

### Cethrin (BA-210)

Following SCI, upstream signaling proteins (axonal growth inhibitors) activate Rho, which on binding to GTP is an activator of Rho-associated coiled-coil kinase (Rho-kinase, Rho/ROCK, serine/threonine kinases; Julian and Olson, 2014). ROCK exists in two isoforms ROCK1 and ROCK2, the expression of latter is 2-fold greater than ROCK1 in CNS (Julian and Olson, 2014). ROCK2 transduces inhibitory signals through modulation of actin cytoskeleton dynamics to block axonal regeneration (Julian and Olson, 2014). Therefore, it is not surprising that transducers Rho-kinases (ROCK1 and ROCK2) have been identified as therapeutic targets in SCI. A number of inactivators of this signaling route have been reportedly used in the clinic and different animal models of SCIs in rats, including Cethrin (BA-210), fasudil and Y-27632. The inhibitors of Rho-kinases promote SCI axonal regeneration and improved locomotor recovery (Lord-Fontaine et al., 2008; McKerracher and Anderson, 2013; Watzlawick et al., 2014).

### Erythropoietin

Erythropoietin (EPO), a glycoprotein, regulates erythropoiesis, and is prescribed for the treatment of anemia (Eschbach et al., 1987; Jelkmann, 2011). The erythropoietin receptor (EPO-R) is expressed in the CNS, including neurons and glial cells (Nagai et al., 2001; Lombardero et al., 2011). This cytokine has long been recognized as a neuroprotectant by inducing non-hematopoietic effects through suppression of inflammation, utilizing anti-apoptogenic pathways, restricting oxidative stress, and improving vascular integrity (Gorio et al., 2002; Mofidi et al., 2011; Simon et al., 2011). Moreover, it possesses neurorestorative properties (Mofidi et al., 2011). Further, EPO has been shown to reduce lesion size, improve structural and functional recovery in the SCI condition, and the benefits have been observed in both animal and human studies (Gorio et al., 2002; King et al., 2007; Simon et al., 2011). However, there are several drawbacks with the use of EPO, such as the increase in hematocrit, which increases blood pressure, and thrombotic complications (Jelkmann, 2011; Lombardero et al., 2011). In order to move away from these negative aspects, investigators have resorted to the use of non-erythropoietic EPO structural variants like carbamylated EPO and asialo-EPO (King et al., 2007; Mofidi et al., 2011; Chen et al., 2015).

### Riluzole

In SCI, persistent stimulation of neuronal voltage-dependent sodium ions channels induces enhanced rates of cell death, arising from acidosis, cellular swelling and glutamatergic toxicity (Wilson and Fehlings, 2014). Riluzole (2-amino-6-[trifluoromethoxy]benzothiazole) is approved by the USA. Food and Drug Administration (FDA) for the treatment of amyotrophic lateral sclerosis (Fehlings et al., 2016). A phase 1 trial of riluzole divulged data on pharmacokinetics, safety, tolerance at the administered dose, and concluded with positive neurological outcomes (Chow et al., 2012). Riluzole is acknowledged to transduce several mechanistic pathways to ameliorate SCI. The benzothiazolic agent mitigates neural glutamatergic-excitotoxicity by inhibiting the interaction between excitatory amino acid glutamate with its consonant glutamate receptor. Also, riluzole blockades voltage-dependent calcium and sodium channels, attenuates apoptosis and inflammation. Collectively, the outcome emanating from these different mechanisms is the sparing of neurological tissue and that has a beneficial effect on functional recovery following SCIs (Hama and Sagen, 2011; Wu et al., 2013; Wilson and Fehlings, 2014). Further, experimental data indicates that riluzole may be a remedy for neuropathic SCI pain too (Hama and Sagen, 2011). The success of riluzole-driven experimental and preclinical studies has been at the cornerstone for progression of the drug onto Phase 2/3 clinical trials for patients with SCI (Fehlings et al., 2016; Table 2).

### Hyperthermia

Application of modest hypothermia (33°C) as a treatment following SCI has managed to garner interest as a consequence of beneficial effects in promoting functional recovery, both in preclinical models and clinical setting (Dietrich, 2009; Lo et al., 2009; Levi et al., 2010). In the rat cervical model of contusive SCI, systemic hypothermia enhanced preservation of tissues (axonal sparing) with prevention of locomotor disability, and augmented skeletal muscle strength (Lo et al., 2009). These promising results have set the stage for application of this technique in clinical trials (Table 2).

### Ibudilast

In co-cultures of neurons and microglial cells, increasing doses of ibudilast, non-selective phosphodiesterase (PDE) inhibitor, decrease neuronal cell death induced by activation of microglia with a combination of lipopolysaccharide (LPS) and IFN-γ. Neuroprotective effect was caused by modulation of inflammatory mediators within activated microglial cells. The Investigators observed reduction in pro-inflammatory cytokines (IL-1β, Il-6 and TNF-γ), suppression of enzymatic activities of iNOS (decrease in synthesis of NO) and NADPH oxidase (reduced production of ROS). In contrast, an augmented secretion of the anti-inflammatory cytokines (IL-10) and neurotrophic factors (nerve growth factor, glia-derived neurotrophic factor, and neurotrophin-4) was observed. Therefore, ibudilast spares neurons from destruction, and reduces the stimulation of microglia (Mizuno et al., 2004). In addition to the preceding beneficial effects, the PDE-antagonist is also effective in controlling neuropathic pain that is presumed to be effective through suppression of activation of innate immune cells (astroglia and microglia; Ledeboer et al., 2007; Walters, 2014).

### Cytotherapy

In different animal models of SCI, a diverse group of cells are under investigation as a therapeutic tool to lower the burden of secondary injury. The cells being transplanted include the glial cells, mesenchymal stromal cells, neural stem cells, pluripotent stem cells, and Schwann cells (El Waly et al., 2014; All et al., 2015; Sabapathy et al., 2015). The successful outcome of cell therapy in experimental models of SCI has raised optimism for these procedures to be translated to the clinic, and hence towards neurobehavioral/ambulatory functional recovery of SCI patients. We illustrate this with two examples of cellular application in SCI scenario.

#### Macrophages

Individual variation in immune responses to comparable type of SCI is a reflection of the presence of distinct macrophage phenotype, either M1 or M2. This thesis was related to the serum levels of pro-inflammatory (M1) or anti-inflammatory/protective (M2) cytokines/chemokines profiles after SCI. The authors suggested the application of this difference can be utilized for the management of acute SCI and neurorehabilitation (Huang et al., 2014). In an earlier study, incubated autologous macrophages promoted locomotion recovery in different rat models of SCI (contusion and transaction). Also, the use of these* in vitro* cultured macrophages for cell-therapy is relatively safe and efficacious as regard to the end-point. Specifically, recovery of sensory/locomotor function in patients, and can be considered as advancement in the treatment of SCI (Knoller et al., 2005).

#### Bone Marrow-Derived Mesenchymal Stem Cells

Intravenously administered rat bone-marrow-derived mesenchymal stem cells (BMMSCs) decreased cavitation volume in BMMSCc-treated rats with contused SCs, and expression of markers for neuronal and glial cells. Functional recovery was more evident when cells were applied within 6 h post-SCI (Osaka et al., 2010). These results are in line with other studies showing significant recovery in locomotor function (Oliveri et al., 2014).

To sum, motivated by positive results from experimental and human studies, investigators have initiated clinical trials based on the aforementioned therapeutic modalities to go beyond the therapeutic administration of MP or in combination with other treatments; and for further information these are cited at http://www.clinicaltrials.gov (USA National Institutes of Health website). In Table 2 are displayed a group of clinical trials that are in progress to examine therapeutic efficacy of cell therapy and pharmacological agents for treatment of SCI.

## Conclusion and Perspectives

To conclude, multi-factorial inflammatory processes are the driving force responsible for the secondary injury following primary insult of SCI. The deleterious actions arising from inflammation are complex, and the mechanisms have been incompletely characterized. As expressed above, diverse responses in the neurovascular compartment of SC trigger secondary injury, including, but not limited to, ischemia reperfusion injury. This milieu leads to activation of leukocytes and SC parenchymal cells, resulting in shift in balance towards inflammatory cells. Constituents of this inflammatory phenotype are amplified oxidative stress, pro-inflammatory cytokines and chemokines, NO system derangement, stimulation of the complement cascade, and impaired endothelial barrier function, eventually leading to axonal and neural degeneration, demyelination and enhanced thrombosis. Collectively, the degree of inflammation is associated with severity and functional decline in SCI patients.

MP, a synthetic glucocorticoid agent, remains the first-line drug of choice for treatment of SCI, and to-date no other medication for SCI therapy has been successfully translated from the bench to the bedside. Therefore, novel therapeutic targets need to be explored. Because of the heterogeneity of secondary injury, a combinatory therapeutic approach may have the desired goal. Application of pharmacological treatment at the earliest will mitigate the deleterious effects of full-blown inflammatory cascades associated with secondary injury. Hence, this action would not only preserve the SC tissue (neural and vascular), but may also aid in functional recovery. However, optimal treatment modalities will only be attained by a profound understanding of inflammatory mechanisms, which are yet to be precisely defined. For this to happen, it is imperative that further studies are conducted in the laboratory as well as in a large set of SCI patient groups at multi-centers, worldwide. It is critical to note that these patient groups ought to encompass age, gender (including pre- and post-menopausal groups) and ethnicity, as well as the three major scourges of humans in 21st century: hypertension, obesity and diabetes. The data obtained from multiple bio-systems through experimental and human investigations will likely help in identifying unique molecular fingerprints as biomarkers, which may be therapeutically targeted in the near future.

## Author Contributions

MAA, TSAS and AHE contributed to the writing. MAA and AHE conceived, designed and revised the manuscript. AHE took care of the final draft.

## Funding

This publication was made possible by Grant #NPRP 4-571-3-171 from the Qatar National Research Fund (a member of Qatar Foundation). The Statements made herein are solely the responsibility of the authors. This Grant was awarded to AHE.

## Conflict of Interest Statement

The authors declare that the research was conducted in the absence of any commercial or financial relationships that could be construed as a potential conflict of interest.
